# Kynurenic acid attenuates experimental allergic rhinitis: Evidence for AhR-associated regulation of mucosal immune homeostasis

**DOI:** 10.1371/journal.pone.0359624

**Published:** 2026-10-01

**Authors:** Naim Gheymoumi, Sevda Shayesteh, Fatemeh Sharifi, Marziyeh Amiri-Andebili, Hediyeh Gheymoumi

**Affiliations:** 1 Department of Pharmacology and Toxicology, Faculty of Pharmacy, Alborz University of Medical Sciences, Karaj, Iran; 2 Student Research Committee, Alborz University of Medical Sciences, Karaj, Iran; Shanghai Jiao Tong University, CHINA

## Abstract

**Background:**

Allergic rhinitis (AR) is a chronic inflammatory airway disease characterized by dysregulated type 2 immune responses and impaired mucosal immune regulation. Kynurenic acid (KYNA), an endogenous tryptophan metabolite with immunomodulatory properties, may influence inflammatory pathways, but its mechanisms in AR remain unclear. This study investigated KYNA in an ovalbumin (OVA)-induced rat model of AR and examined the role of aryl hydrocarbon receptor (AhR) signaling.

**Methods:**

Rats were assigned to control, AR, vehicle, KYNA, desloratadine, and KYNA + AhR antagonist (CH223191) groups. Following OVA sensitization and challenge, behavioral responses, serum inflammatory markers, nasal AhR-associated proteins, regulatory cytokines, and histopathological changes in nasal and splenic tissues were evaluated.

**Results:**

OVA induced typical allergic responses, including increased sneezing and nasal scratching, elevated serum IgE, IL-4, and IL-6, and marked inflammatory changes in nasal and splenic tissues. KYNA significantly reduced behavioral symptoms, systemic inflammatory mediators, epithelial damage, and inflammatory infiltration, with systemic effects comparable to desloratadine but greater improvement in nasal scratching and splenic histopathology. KYNA restored nasal AhR and CYP1A1 expression and increased IL-10 and TGF-β, whereas CH223191 partially attenuated these effects, indicating involvement of AhR signaling. Desloratadine showed limited effects on local regulatory cytokines. Histopathological analysis confirmed improved nasal and splenic architecture following KYNA treatment.

**Conclusion:**

In conclusion, KYNA attenuates experimental AR through systemic anti-inflammatory effects and restoration of mucosal immune regulation. AhR signaling contributes to these effects, particularly in CYP1A1 and cytokine regulation, while additional mechanisms may also be involved. These findings support KYNA as a potential immunometabolic modulator in allergic airway disease.

## 1. Introduction

Allergic rhinitis (AR) is a chronic inflammatory disorder of the upper airway characterized by nasal congestion, rhinorrhea, sneezing, and nasal pruritus following exposure to environmental allergens [1]. The disease affects a substantial proportion of the global population and contributes significantly to impaired quality of life, sleep disturbance, reduced work productivity, and healthcare utilization [2,3]. Although antihistamines and intranasal corticosteroids provide symptomatic relief in many patients, persistent inflammation and disease recurrence remain important clinical challenges [4,5].

The pathogenesis of AR involves a dysregulated immune response to otherwise innocuous environmental antigens. Sensitization results in allergen-specific IgE production, activation of mast cells and other effector immune cells, and release of inflammatory mediators that drive characteristic symptoms [6]. In addition to classical type 2 immune responses, increasing evidence indicates that defective immunoregulatory mechanisms contribute to the persistence of allergic inflammation and impaired mucosal homeostasis [7,8]. Consequently, growing attention has focused on endogenous pathways that regulate immune responses within barrier tissues [8,9].

The aryl hydrocarbon receptor (AhR) is a ligand-activated transcription factor expressed in epithelial cells and numerous immune cell populations [10]. Beyond its traditional role as a sensor of environmental compounds, AhR has emerged as an important regulator of immune homeostasis, epithelial barrier function, and inflammatory responses at mucosal surfaces [10,11]. Experimental studies have demonstrated that AhR signaling influences cytokine production, immune cell differentiation, and tissue repair processes within the respiratory tract [12]. Dysregulation of AhR-associated pathways has been implicated in several inflammatory and allergic disorders, including asthma and allergic airway inflammation [13,14].

Kynurenic acid (KYNA) is an endogenous metabolite generated through the kynurenine pathway of tryptophan metabolism. In addition to its established neurobiological functions, KYNA exhibits anti-inflammatory and immunomodulatory properties in multiple experimental models [15,16]. Previous studies have identified KYNA as a ligand capable of interacting with AhR signaling pathways [17]. However, KYNA is a pleiotropic molecule whose biological effects may also involve additional mechanisms, including interactions with G protein-coupled receptor 35 (GPR35), modulation of oxidative stress, and regulation of inflammatory signaling pathways [18,19]. The relative contribution of AhR-associated signaling to the anti-inflammatory actions of KYNA in allergic rhinitis remains incompletely understood.

Among the mediators involved in immune regulation, interleukin-10 (IL-10) and transforming growth factor-beta (TGF-β) play central roles in maintaining immune tolerance and limiting excessive inflammatory responses [20–22]. Reduced expression of these cytokines has been reported in allergic disorders and may contribute to impaired mucosal immune regulation. Whether KYNA influences local regulatory cytokine responses through AhR-associated mechanisms in allergic rhinitis has not been fully investigated.

Therefore, the present study evaluated the effects of KYNA in an ovalbumin-induced rat model of allergic rhinitis. To explore the potential involvement of AhR-associated signaling, the AhR antagonist CH223191 was employed. Behavioral symptoms, systemic inflammatory biomarkers, histopathological alterations, and local nasal levels of AhR, CYP1A1, IL-10, and TGF-β were assessed. Therefore, the present study aimed to investigate whether KYNA attenuates the clinical, inflammatory, and histopathological manifestations of experimental allergic rhinitis and to determine whether the effects of KYNA are mediated, at least in part, through AhR-associated signaling.

## 2. Materials and methods

### 2.1 Animals and experimental design

Forty-eight adult male Wistar rats (250–350 g) were used in this study. Animals were housed under standard laboratory conditions (12 h light/dark cycle, 22 ± 2°C) with ad libitum access to food and water. All experimental procedures were approved by the Institutional Animal Care and Use Committee of Alborz University of Medical Sciences(Ethics Code: IR.ABZUMS.AEC.1402.006). Animals were monitored daily throughout the experimental period for general health status, body weight, food and water intake, mobility, grooming behavior, and signs of pain or distress. Humane endpoints were predefined before study initiation and included severe lethargy, persistent body weight loss exceeding 20% of baseline, impaired mobility, inability to access food or water, or severe respiratory distress. Animals meeting any of these criteria would have been euthanized immediately to minimize suffering. No animals reached the predefined humane endpoints or died spontaneously during the study.

The sample size was determined a priori using G*Power software (version 3.1.9.7) for a one-way analysis of variance (ANOVA) involving six experimental groups. The calculation was performed assuming an effect size of f = 0.60, an alpha error probability of 0.05, and a statistical power of 80% (1 − β = 0.80). Under these assumptions, the minimum required sample size was 42 animals (7 animals per group). To allow for potential attrition and to ensure adequate numbers for the planned analyses, 8 animals were allocated to each group, resulting in a total of 48 rats.

Animals were randomly allocated into six experimental groups (n = 8 per group): (1) Control, (2) allergic rhinitis (AR), (3) vehicle (Al(OH)_3_), (4) KYNA, (5) desloratadine, and (6) KYNA + CH223191. The Control group provided baseline values, whereas the AR group represented the disease model. The vehicle group was included to determine whether administration of the vehicle used for treatment influenced the measured outcomes independently of the active treatments. KYNA was administered to evaluate its effects on experimental AR, while desloratadine served as a reference anti-allergic treatment. CH223191 was administered in combination with KYNA as a pharmacological AhR antagonist to investigate the potential involvement of AhR signaling in KYNA-mediated effects, particularly for the local nasal molecular endpoints. Accordingly, the CH223191 group was not included in all experimental assessments.

### 2.2 Induction of allergic rhinitis

Allergic rhinitis was induced using ovalbumin (OVA; Sigma-Aldrich, USA). Animals in sensitized groups received intraperitoneal injections of OVA (0.1 mg/mL) emulsified with aluminum hydroxide [Al(OH)_3_] (1 mg/mL) in normal saline on alternate days from Day 0 to Day 14 to establish systemic sensitization [23].

Following sensitization, nasal allergic inflammation was induced by intranasal OVA exposure during the challenge phase as described below.

### 2.3 Experimental timeline and treatment protocol

From Day 15 to Day 29, animals received once-daily pharmacological treatments as follows: KYNA (10 mg/kg/day, intraperitoneally), desloratadine (5 mg/kg/day, orally), or saline in corresponding control groups. In the KYNA + CH223191 group, CH223191 (10 mg/kg/day, intraperitoneally) was administered 30 min prior to KYNA administration each day.

The selected dosages were designed to assess the therapeutic effects of subchronic modulation of the kynurenine pathway. Daily administration of KYNA (10 mg/kg) and desloratadine (5 mg/kg) over 14 days was intended to maintain stable systemic exposure, thereby enabling detection of downstream transcriptional changes in the AhR–CYP1A1 signaling axis and their subsequent impact on inflammatory mediator regulation [24].

### 2.4 Intranasal challenge and behavioral assessment

On Day 30, all animals were challenged intranasally with OVA (0.5 μL per nostril) [23]. Immediately after administration, animals were individually placed in observation chambers, and allergic behaviors were recorded for 40 min by an observer blinded to group allocation. Sneezing frequency and nasal scratching behavior were quantified. Nasal scratching was defined as repetitive forepaw contact with the nasal region.

### 2.5 Sample collection

At the end of the experiment, animals were deeply anesthetized with ketamine (60 mg/kg) and xylazine (10 mg/kg). Blood samples were collected by cardiac puncture under deep anesthesia, centrifuged at 3500 rpm for 10 min at 4°C, and serum was stored at −80°C until analysis. The nasal mucosa and spleen were immediately harvested for subsequent analyses. Spleen weight was recorded, and the spleen-to-body weight ratio was calculated as an index of systemic immune activation. Tissue samples were either fixed in 10% neutral-buffered formalin for histological evaluation or snap-frozen for subsequent biochemical analyses.

### 2.6 ELISA and tissue protein quantification

Serum concentrations of IgE, IL-4, and IL-6 were measured using commercially available enzyme-linked immunosorbent assay (ab157736, ab100770, ab234570, Abcam) ELISA kits according to the manufacturers’ instructions. Blood samples were collected via cardiac puncture, allowed to clot at room temperature, and centrifuged at 3500 rpm for 10 min at 4°C to obtain serum. All samples and standards were analyzed in duplicate using a microplate-based assay, and optical density was measured at the 450 nm wavelength specified by each kit protocol.

Levels of AhR (abx353504, Abbexa), CYP1A1 (abx256388, Abbexa), IL-10 (ab100765, abcam) and TGF-β (MBS160117, MyBioSource) in nasal tissue homogenates were quantified using commercially available ELISA kits according to the manufacturers’ instructions. Briefly, frozen nasal tissues were homogenized in ice-cold phosphate-buffered saline containing protease inhibitors and centrifuged at 5000 × g for 15 min at 4°C to obtain clear supernatants. All samples and standards were assayed in duplicate, and optical density was measured using a microplate reader at the recommended wavelength.

Total protein concentration in each sample was determined using a bicinchoninic acid (BCA) assay to account for variations in tissue mass and extraction efficiency. Concentrations of target proteins and cytokines were normalized to total protein content and expressed as pg/mg protein for AhR, IL-10, and TGF-β, and as ng/mg protein for CYP1A1.

### 2.7 Histopathological evaluation

Nasal and splenic tissues were fixed in 10% neutral-buffered formalin, embedded in paraffin, and sectioned at 5–10 μm thickness. Sections were stained with hematoxylin and eosin (H&E). Histopathological evaluation was performed by a blinded investigator. For each animal, multiple non-overlapping fields from at least three sections were examined under light microscopy at ×40 magnification, and mean scores were calculated.

Nasal mucosal inflammation was graded on a scale of 0–3 as follows: **0 (normal)**, no inflammatory cell infiltration, intact epithelial structure, and no edema; **1 (mild)**, slight inflammatory infiltration with largely preserved epithelium and minimal edema; **2 (moderate)**, moderate inflammatory infiltration with focal epithelial disruption and evident edema; **3 (severe)**, dense inflammatory infiltration with marked epithelial damage and pronounced edema [23].

Splenic histopathological alterations were also scored on a 0–3 scale as follows: **0 (normal)**, preserved white pulp and red pulp architecture; **1 (mild)**, slight white pulp or follicular enlargement with minimal architectural changes; **2 (moderate)**, clear white pulp hyperplasia with partial disruption of normal organization; **3 (severe)**, marked white pulp hyperplasia with extensive architectural disruption and loss of normal splenic structure [23].

### 2.8 Statistical analysis

Data are presented as mean ± standard error of the mean (SEM). Statistical analyses were performed using GraphPad Prism 8.0. Normality was assessed using the Shapiro–Wilk test. Group comparisons were performed using one-way ANOVA followed by Tukey’s post hoc test. P < 0.05 was considered statistically significant.

## 3. Results

### 3.1 Effects of KYNA and AhR antagonism on allergic behavioral responses

OVA sensitization and challenge produced characteristic manifestations of allergic rhinitis, with significantly increased nasal scratching and sneezing frequencies compared with the control group (Fig 1A and 1B; P < 0.01 and P < 0.001, respectively). No significant differences were observed between the control and vehicle groups, indicating that administration of aluminum hydroxide alone did not affect behavioral responses (P > 0.05).

**Fig 1:**
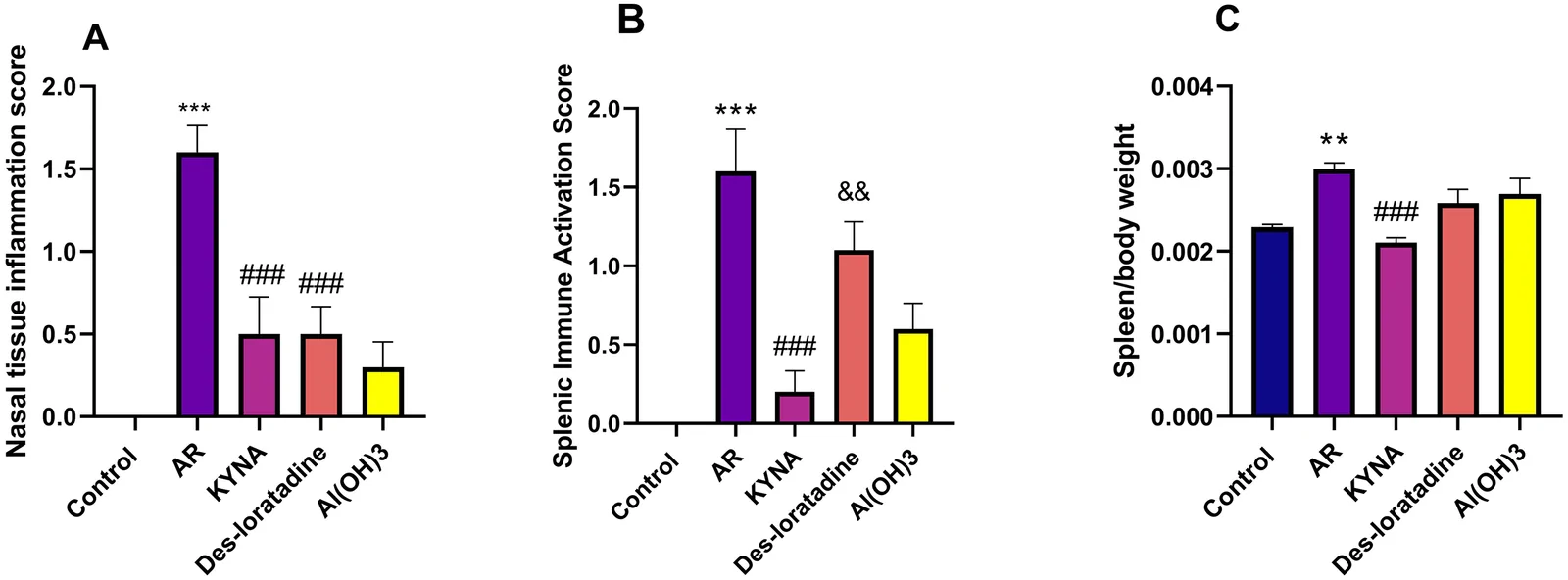
Effects of KYNA and pharmacological AhR blockade on allergic behavioral responses following ovalbumin challenge. (A) Nasal itching score and (B) sneezing frequency across the six experimental groups. Data are presented as mean ± SEM (n = 8). Statistical significance is indicated as follows: **P < 0.01 and ***P < 0.001 vs. Control; #P < 0.05 and ###P < 0.001 vs. AR; &P < 0.05, &&P < 0.01, and &&&P < 0.001 vs. KYNA.

KYNA treatment significantly reduced nasal scratching and sneezing compared with the untreated AR group (P < 0.001). Desloratadine significantly reduced sneezing frequency (P = 0.029), whereas its effect on nasal scratching was less pronounced than that of KYNA (P < 0.01 vs. KYNA). Co-administration of the AhR antagonist CH223191 significantly attenuated the beneficial effects of KYNA on nasal scratching and sneezing compared with KYNA alone (P = 0.038 and P < 0.001, respectively). For several behavioral endpoints, the CH223191-treated group did not differ significantly from the untreated AR group, indicating attenuation of the KYNA-associated behavioral improvement.

### 3.2 Effects of KYNA on systemic inflammatory biomarkers

Serum concentrations of IgE, IL-4, and IL-6 were significantly elevated in the AR group compared with the control group (P < 0.001 for all; Fig 2A–2C), confirming successful induction of systemic allergic inflammation. The vehicle group showed no significant differences from the control group for these biomarkers (P > 0.05).

**Fig 2:**
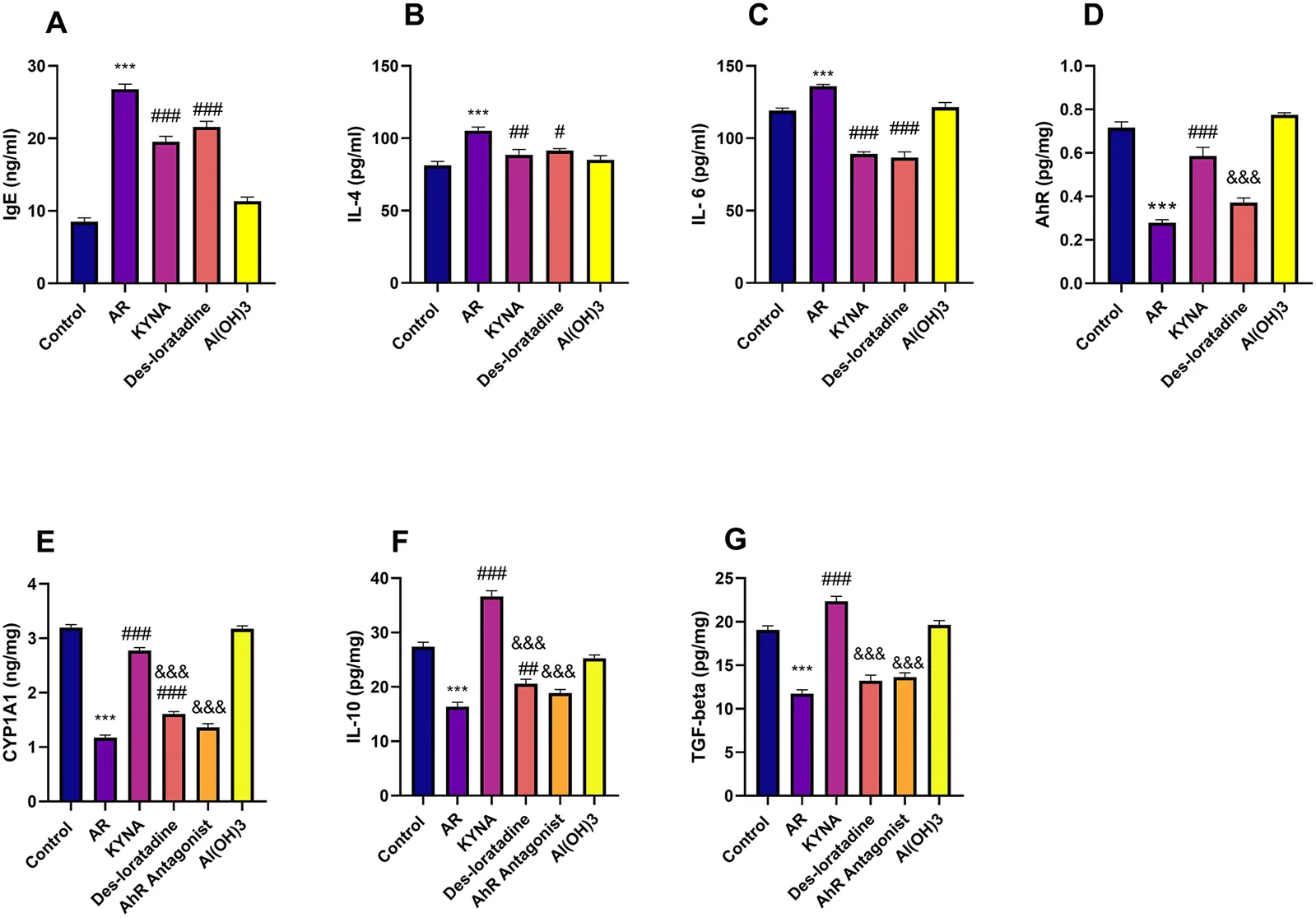
Effects of KYNA and AhR antagonism on systemic inflammatory biomarkers and nasal mucosal protein expression. Serum levels of (A) IgE, (B) IL-4, and (C) IL-6 are shown. Nasal tissue homogenate levels of (D) AhR, (E) CYP1A1, (F) IL-10, and (G) TGF-β are also presented. Data are expressed as mean ± SEM (n = 8). ***P < 0.001 vs. Control; #P < 0.05, ##P < 0.01, and ###P < 0.001 vs. AR; &P < 0.05 and &&&P < 0.001 vs. KYNA.`.

KYNA treatment significantly reduced serum IgE, IL-4, and IL-6 concentrations compared with the untreated AR group (P < 0.001, P = 0.0013, and P < 0.001, respectively). Desloratadine significantly reduced IgE, IL-4, and IL-6 levels compared with the AR group (P < 0.001, P = 0.0105, and P < 0.001, respectively). No significant differences were observed between the KYNA and desloratadine groups for any of the measured systemic inflammatory biomarkers (P > 0.05). CH223191 was not evaluated in the systemic biomarker and histopathology analyses, as this group was included specifically to assess AhR-dependent mechanisms at the local nasal mucosal level.

### 3.3 Effects of KYNA on nasal AhR-associated proteins and regulatory cytokines

To evaluate local mucosal responses, AhR, CYP1A1, IL-10, and TGF-β levels were measured in nasal tissue homogenates (Fig 2D–2G). Nasal AhR and CYP1A1 levels were significantly lower in the AR group than in the control group (P < 0.001). The vehicle group showed no significant differences from the control group (P > 0.05). KYNA treatment significantly increased both AhR and CYP1A1 levels compared with the untreated AR group (P < 0.001). Desloratadine also significantly increased CYP1A1 levels (P < 0.001); however, the increases in both AhR and CYP1A1 were greater in the KYNA group than in the desloratadine group (P < 0.001). Co-administration of CH223191 significantly attenuated the KYNA-induced increase in CYP1A1, reducing its level toward that observed in the AR group (P < 0.001) (Fig 2D and 2E). Similarly, IL-10 and TGF-β levels were significantly decreased in the AR group compared with the control group (P < 0.001). KYNA treatment significantly increased both cytokines compared with the untreated AR group (P < 0.001). Desloratadine significantly increased IL-10 (P = 0.0075) but did not significantly affect TGF-β compared with the AR group. IL-10 and TGF-β levels were both significantly higher in the KYNA group than in the desloratadine group (P < 0.001 for both comparisons). Co-administration of CH223191 significantly attenuated the KYNA-induced increases in IL-10 and TGF-β, with both cytokines reduced toward the levels observed in the AR group (P < 0.001 for both) (Fig 2F and 2G).

### 3.4 Histopathological findings

#### 3.4.1 Histopathological evaluation of nasal tissue.

Histopathological analysis of the nasal mucosa revealed significant differences among the experimental groups. The AR group showed a significantly higher inflammatory score than both the control and vehicle groups (P < 0.001) (Fig 3A). Histological examination revealed epithelial disruption, mucosal edema, and inflammatory cell infiltration in the AR group (Fig 4B).

**Fig 3:**
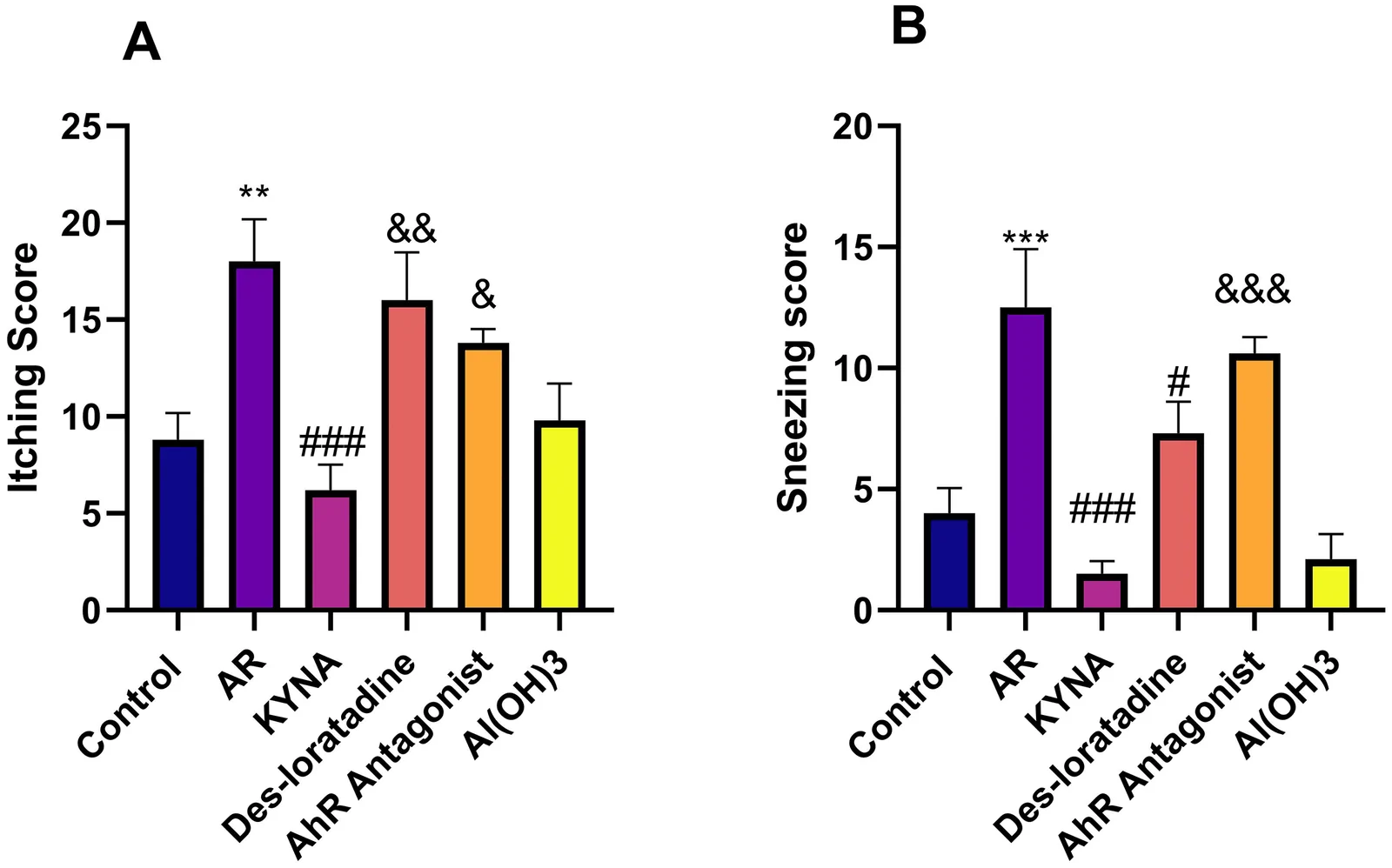
Histopathological scoring and organ index analysis across experimental groups. (A) Nasal mucosal inflammation score, (B) splenic histopathological alteration score, and (C) spleen-to-body weight ratio. Data are presented as mean ± SEM (n = 8). **P < 0.01 and ***P < 0.001 vs. Control; ###P < 0.001 vs. AR; &&P < 0.01 vs. KYNA.

**Fig 4:**
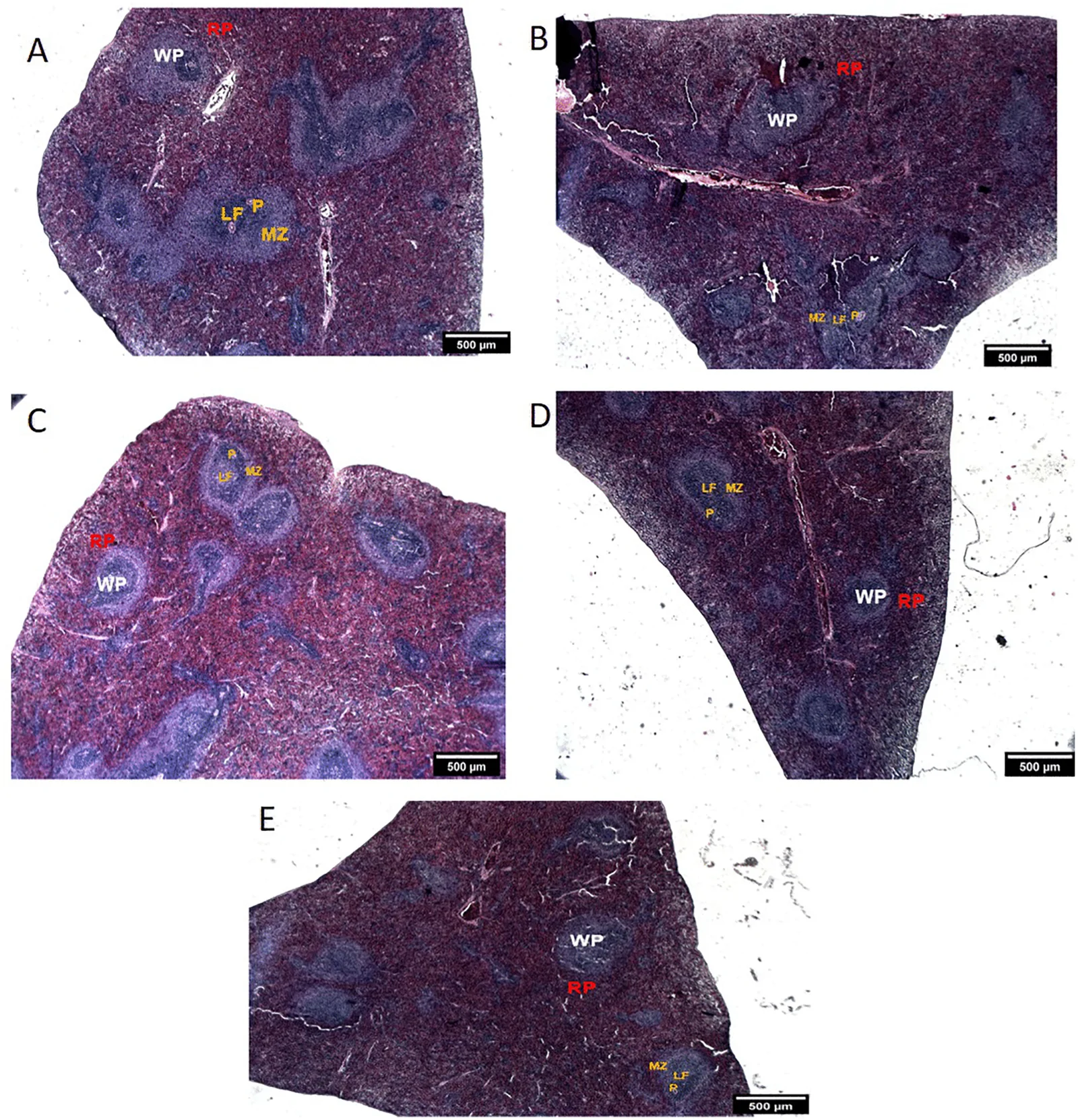
Representative hematoxylin and eosin-stained nasal mucosal sections from all experimental groups. (Edema: o; eosinophilic infiltrate: arrows; abnormal epithelium: asterisk). The AR group showed marked epithelial disruption, submucosal edema, and inflammatory cell infiltration compared with the control group. KYNA and desloratadine treatment attenuated these histopathological alterations. Samples include (A) Control, (B) AR, (C) Al(OH)_3_, (D) KYNA, and (E) desloratadine groups. All images were obtained at ×40 magnification.

KYNA treatment significantly reduced the nasal inflammatory score compared with the AR group (P < 0.001) (Figs 3A and 4D). Desloratadine produced a similar significant reduction (P < 0.001), and no significant difference was observed between the KYNA and desloratadine groups (P > 0.05), indicating comparable effects on nasal histopathological changes. No significant difference was observed between the vehicle and control groups (P > 0.05), indicating that aluminum hydroxide administration alone did not induce detectable histopathological changes in the nasal mucosa (Figs 3A and 4C).

#### 3.4.2 Histopathological evaluation of splenic tissue.

Histological examination of the spleen revealed significant alterations in the AR group compared with both the control and vehicle groups (P < 0.001) (Fig 3B). These changes included disruption of normal splenic architecture and alterations within white pulp regions (Fig 5B).

**Fig 5:**
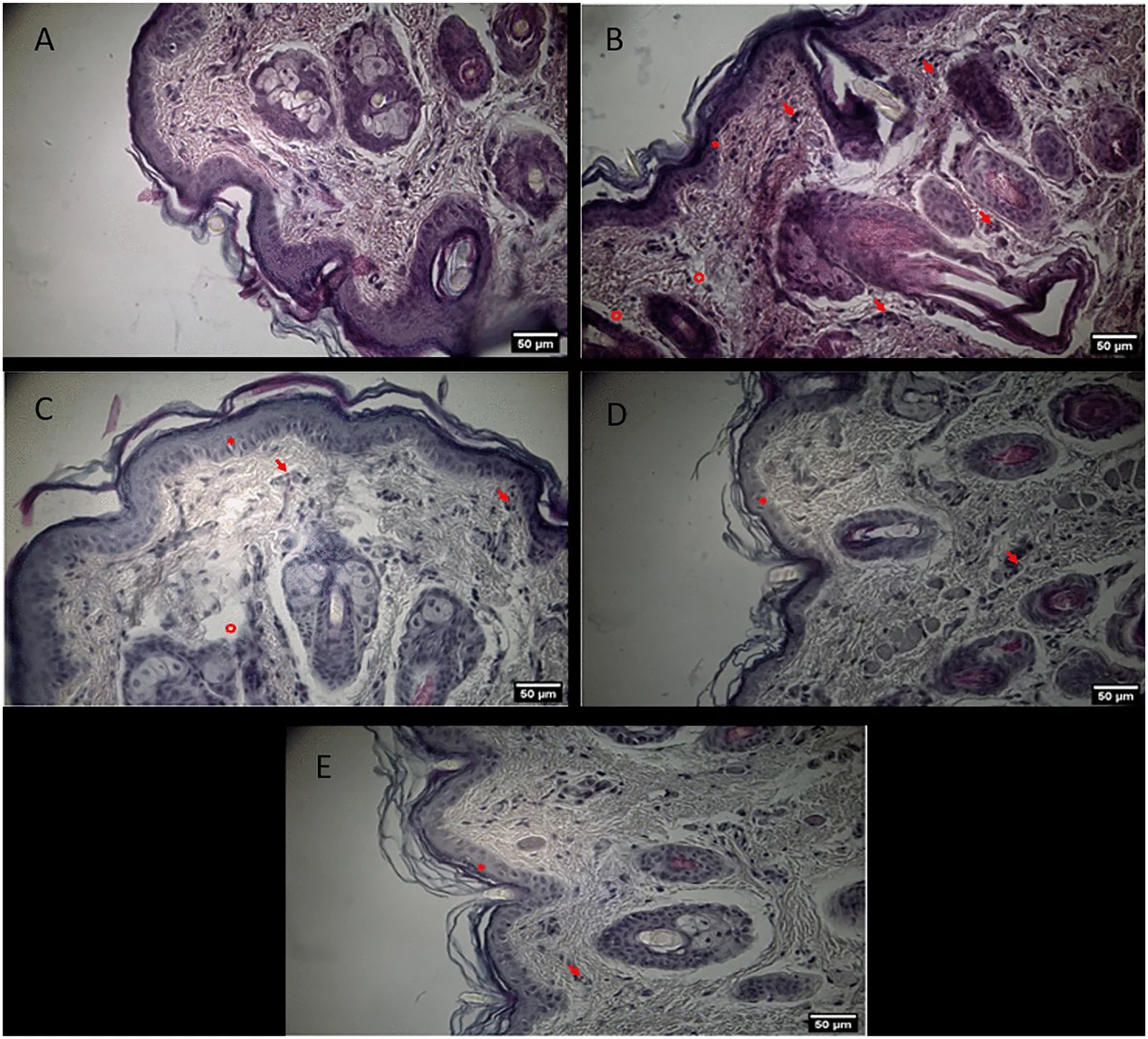
Histopathological examination of splenic tissue stained with hematoxylin and eosin. (Red pulp: RP; white pulp: WP; periarteriolar lymphocyte sheath: PALS; lymphoid follicles: LF; marginal zones: MZ; abnormal architecture: asterisk). The AR group demonstrated disruption of normal splenic architecture compared with controls. KYNA partially restored splenic organization, whereas desloratadine showed limited improvement. Samples include (A) Control, (B) AR, (C) Al(OH)_3_, (D) KYNA, and (E) desloratadine groups. All images were obtained at ×40 magnification.

KYNA treatment significantly reduced the splenic histopathological score compared with the AR group (P < 0.001) (Figs 3B and 5D), indicating partial restoration of normal splenic architecture. In contrast, desloratadine did not significantly improve the splenic histopathological score compared with the AR group (P > 0.05) (Figs 3B and 5E). The splenic histopathological score was significantly lower in the KYNA group than in the desloratadine group (P = 0.005). No significant difference was observed between the vehicle and control groups (P > 0.05), indicating that the vehicle did not produce detectable histopathological alterations in the spleen (Figs 3B and 5C).

#### 3.4.3 Spleen-to-body weight ratio (spleen index).

OVA sensitization and challenge resulted in a significant increase in the spleen-to-body weight ratio in the untreated AR group compared with healthy controls (P = 0.0022; Fig 3C).

Treatment with KYNA significantly reduced the spleen index compared with the untreated AR group (P < 0.001; Fig 3C). In contrast, treatment with desloratadine did not significantly reduce the spleen-to-body weight ratio compared with the AR group (P > 0.05). No significant difference was observed between the vehicle (Al(OH)_3_) and control groups (P > 0.05).

## 4. Discussion

The present study demonstrates that kynurenic acid (KYNA) attenuates key features of ovalbumin (OVA)-induced allergic rhinitis (AR), including behavioral symptoms, systemic inflammatory responses, and histopathological changes. KYNA also increased the nasal expression of AhR-associated proteins and the regulatory cytokines IL-10 and TGF-β. Several of these effects were reduced by pharmacological inhibition of the aryl hydrocarbon receptor (AhR), indicating that AhR signaling contributes to KYNA-mediated immunomodulation.

OVA sensitization and challenge induced expected allergic inflammation, including increased sneezing and nasal scratching, elevated serum IgE, IL-4, and IL-6, and marked inflammatory changes in nasal and splenic tissues. These findings align with established OVA-induced allergic airway disease models and confirm a type 2-dominant inflammatory response [25,26]. The associated reduction in local regulatory cytokines and histopathological evidence of epithelial disruption and inflammatory-cell infiltration further indicate an imbalance between inflammatory activation and mucosal immune regulation.

These findings are consistent with the established pathophysiology of allergic rhinitis, which involves coordinated interactions between the nasal epithelium, innate and adaptive immune responses, and local regulatory mechanisms. Disruption of epithelial barrier integrity can increase mucosal permeability to allergens and promote the release of epithelial-derived mediators that facilitate type 2 inflammation [27]. Allergen exposure subsequently promotes Th2-associated cytokine responses, including IL-4, which supports B-cell class switching and IgE production [28]. Allergen-specific IgE binds to FcεRI on mast cells, and subsequent allergen-induced cross-linking triggers mast-cell activation and release of mediators that contribute to nasal itching, sneezing, vascular changes, and mucosal edema. These responses are accompanied by recruitment and activation of inflammatory cells, including eosinophils and other leukocyte populations, which further amplify local tissue inflammation [29]. At the same time, impaired local regulatory mechanisms, including IL-10- and TGF-β-mediated immunoregulation, may contribute to persistence of the inflammatory response [30]. Thus, AR is not solely a consequence of excessive type 2 immunity but reflects an imbalance between epithelial integrity, inflammatory activation, and mechanisms responsible for maintaining mucosal immune tolerance.

KYNA significantly reduced behavioral and systemic inflammatory indices. Decreased IgE and IL-4 indicate suppression of Th2 responses, while reduced IL-6 reflects broader anti-inflammatory effects [31–33]. KYNA and desloratadine showed comparable effects on circulating markers, suggesting similar systemic immunomodulation. However, KYNA produced greater reductions in nasal scratching and stronger effects on local regulatory pathways, including AhR, CYP1A1, IL-10, and TGF-β. In contrast, desloratadine mainly affected downstream inflammatory outcomes with limited impact on mucosal regulatory markers, indicating that KYNA additionally promotes local immune homeostasis.

A key finding was the reduction of nasal AhR and CYP1A1 expression after OVA challenge. AhR is a central regulator of barrier immunity, integrating environmental and metabolic signals to maintain mucosal balance, and its downregulation is linked to impaired epithelial function and heightened inflammation [34]. KYNA restored AhR and CYP1A1 expression, while CH223191 co-treatment reduced CYP1A1 induction and attenuated KYNA’s protective effects. As CYP1A1 is a canonical AhR target, these results support involvement of AhR signaling [35]. However, because receptor activation and transcriptional activity were not directly assessed, these findings indicate pathway involvement rather than definitive AhR agonism.

KYNA also increased IL-10 and TGF-β, key cytokines for mucosal immune tolerance. IL-10 suppresses antigen presentation and inflammatory cytokine production, while TGF-β supports immune regulation, epithelial repair, and regulatory T-cell maintenance [36,37]. Their reduction in AR reflects impaired local immune control, whereas their restoration by KYNA indicates enhanced mucosal regulatory function.

AhR inhibition partially reduced KYNA-induced IL-10 and TGF-β increases, suggesting that AhR contributes but does not fully mediate their regulation. The remaining effects imply additional pathways, including potential involvement of GPR35, a proposed KYNA receptor linked to inflammatory and epithelial regulation, as well as KYNA’s antioxidant and redox-modulating properties [18]. These mechanisms were not directly tested but may explain residual effects under AhR blockade.

Overall, KYNA appears to act as an endogenous regulator of mucosal immune homeostasis rather than solely an anti-inflammatory agent. By modulating AhR signaling, KYNA restores disrupted regulatory pathways, while AhR-independent mechanisms such as GPR35 or redox signaling may provide complementary effects [38,39]. This dual action likely explains KYNA’s broader impact on local immune regulation compared with desloratadine, despite similar systemic effects.

Histopathologically, KYNA reduced epithelial damage, inflammatory infiltration, and mucosal edema in nasal tissues and improved splenic abnormalities associated with systemic immune activation. These findings support the functional relevance of the molecular and cytokine changes observed at both local and systemic levels and are consistent with previous reports describing immunomodulatory effects of KYNA. Exogenous KYNA has been shown to exhibit low cytotoxicity toward murine splenocytes and good in vivo tolerability, while reducing activation-induced inflammatory responses in vitro and ex vivo [40].

Limitations include reliance on pharmacological rather than genetic AhR inhibition, possible off-target effects of CH223191, indirect assessment of receptor activation, lack of identification of IL-10 and TGF-β cellular sources, and absence of direct evaluation of GPR35 and oxidative stress pathways. Only one KYNA dose and regimen were tested. Future studies should include genetic models, cell-specific analyses, and direct signaling assessments to clarify pathway contributions.

In summary, KYNA attenuates allergic rhinitis through systemic anti-inflammatory effects and restoration of local mucosal regulation. AhR signaling plays a key role, particularly in CYP1A1 expression and IL-10/TGF-β regulation, while additional pathways may contribute. These findings support further investigation of KYNA and related immunometabolic mediators in allergic airway disease.

## 5. Conclusions

KYNA attenuated key features of allergic rhinitis, including behavioral symptoms, systemic inflammatory responses, and histopathological alterations, while also increasing nasal expression of AhR-associated proteins and regulatory cytokines in an OVA-induced rat model. The partial reversal of these effects by the AhR antagonist CH223191 indicates that AhR-associated signaling contributes substantially to the immunomodulatory actions of KYNA. Overall, these findings support a potential role for endogenous AhR ligands in regulating mucosal immune responses and highlight KYNA as a candidate modulator of allergic airway inflammation.

## Supporting information

S1 Data Data. (XLSX)
